# Feline Morbillivirus Infection in Domestic Cats: What Have We Learned So Far?

**DOI:** 10.3390/v13040683

**Published:** 2021-04-15

**Authors:** Eliana De Luca, Giuseppe Andrea Sautto, Paolo Emidio Crisi, Alessio Lorusso

**Affiliations:** 1 Istituto Zooprofilattico Sperimentale dell’Abruzzo e Molise (IZSAM), 64100 Teramo, Italy; elianadeluca9@gmail.com; 2 Center for Vaccines and Immunology, University of Georgia, Athens, GA 30605, USA; gasautto@uga.edu; 3 Faculty of Veterinary Medicine, Veterinary University Hospital, University of Teramo, 64100 Teramo, Italy; pecrisi@unite.it

**Keywords:** feline morbillivirus, genetic heterogeneity, epidemiology, kidney disease, tropism, diagnosis

## Abstract

Feline morbillivirus (FeMV) was identified for the first time in stray cats in 2012 in Hong Kong and, since its discovery, it was reported in domestic cats worldwide. Although a potential association between FeMV infection and tubulointerstitial nephritis (TIN) has been suggested, this has not been proven, and the subject remains controversial. TIN is the most frequent histopathological finding in the context of feline chronic kidney disease (CKD), which is one of the major clinical pathologies in feline medicine. FeMV research has mainly focused on defining the epidemiology, the role of FeMV in the development of CKD, and its in vitro tropism, but the pathogenicity of FeMV is still not clear, partly due to its distinctive biological characteristics, as well as to a lack of a cell culture system for its rapid isolation. In this review, we summarize the current knowledge of FeMV infection, including genetic diversity of FeMV strains, epidemiology, pathogenicity, and clinicopathological findings observed in naturally infected cats.

## 1. Introduction

Paramyxoviruses constitute a large group of viruses that are responsible for important diseases in humans and animals [1]. According to the International Committee on Taxonomy of Viruses (ICTV, https://talk.ictvonline.org, accessed on 14 April 2021), the family *Paramyxoviridae* is divided into four subfamilies (*Metaparamyxovirinae*, *Avulavirinae*, *Orthoparamyxovirinae* and *Rubulavirinae*) and within the *Orthoparamyxovirinae* subfamily, eight genera have been established (*Aquaparamyxovirus*, *Ferlavirus*, *Jeilongvirus*, *Henipavirus*, *Morbillivirus*, *Narmovirus*, *Respirovirus*, and *Salemvirus*). The *Morbillivirus* genus includes highly infectious viruses, such as canine distemper virus (CDV), peste-des-petits-ruminants virus, cetacean morbillivirus, and measles virus (MeV), which can cause severe and occasionally fatal systemic diseases [2,3,4,5]. In the last decade, the genus *Morbillivirus* has received growing attention, due to the recent discovery of a new feline morbillivirus (FeMV) in stray cats from Hong Kong and Mainland China [6]. The first case–control study [6] has proposed an association of FeMV infection with chronic tubulointerstitial nephritis (TIN), the most frequent histopathological finding in feline chronic kidney disease (CKD) [7]. CKD affects approximately 30% of cats older than 10 years and represents the major clinical complication in this age group [8,9]. Following its discovery, FeMV was also described in other countries including Japan [10,11,12,13], USA [14], Turkey [15], Brazil [16], Thailand [17], Italy [18,19,20,21,22,23], United Kingdom [24], Germany [25,26], Malaysia [27], and Mainland China [28]. While several studies suggested an association between FeMV infection and kidney disease in infected cats [6,10,12,13,18,26,29], many others have investigated, but not demonstrated, the causative role of FeMV in the pathogenesis of feline CKD [14,15,16,17,21,22,23,24,28]. Thus, the aim of this review is to provide a summary of the current knowledge regarding FeMV, focusing on epidemiology, pathogenesis, and clinicopathological aspects of FeMV infection. Virus isolation and molecular and serological methods developed for FeMV diagnosis are also covered in this review.

## 2. FeMV Is Classified in the Genus *Morbillivirus* within the *Paramyxoviridae* Family

The FeMV genome is a single-stranded, negative-sense RNA with a size of 16,050 bases, representing the largest genome among all morbilliviruses [6]. In members belonging to the *Paramyxoviridae* family, all genes are separated by untranslated regions, which include stop signals at the end of the upstream gene and start signals for the expression of downstream genes. There are also conserved sequences at the 3′ and 5′ ends of the genome, which are used as promoters by the RNA-dependent RNA polymerase. At the 3′ end, is a leader sequence (55 nucleotides), which represents the genome promoter for the synthesis of viral messenger RNA or full-length antigenome (positive-sense RNA). Similarly, at the 5′ end of the genome, there is a conserved long trailer sequence including the antigenome promoter, which is responsible for the production of full-length genomic RNA (negative-sense) from the antigenome [30]. In FeMV, this trailer sequence is much longer than that of other morbilliviruses: these typically consist of only 40–41 nucleotides, while the genome of FeMV has a 5′ trailer sequence of 400 nucleotides [6,19]. Within the family, such long trailer sequences have been described only in avian paramyxoviruses (APMV), specifically in APMV-3 (707 nt) [31] and APMV-5 (552 nt) [32]. As for other paramyxoviruses, the genome of FeMV conforms to the “rule of six”, since each nucleocapsid protein (N) monomer encapsidates six nucleotides of RNA [30]. Moreover, as a member of the *Morbillivirus* genus, FeMV contains six non-overlapping genes in the following order N–P/V/C–M–F–H–L, encoding eight structural and non-structural proteins [19]. The two non-structural proteins V and C are encoded within the P open reading frame by RNA editing and alternative translation initiation, respectively [30]. In morbilliviruses, the six encoded structural proteins include a matrix protein (M), two RNA-polymerase-associated proteins (the phosphoprotein P and the large protein L), a nucleocapsid protein (N) and two glycoproteins (the hemagglutinin H and the fusion protein F) [33]. The genome is encapsidated in the nucleocapsid, which includes proteins N, P, and L. The latter proteins along with the viral RNA form a ribonucleoprotein complex that is responsible for the transcription and the replication steps [34]. Externally, these viruses are enveloped by a host plasma membrane-derived lipid bilayer acquired during the budding process. The M protein represents a bridge between the envelope and the nucleocapsid and is involved in virus particle assembly and budding [35,36]. During virus biogenesis, the F glycoprotein precursor is cleaved into the biologically active and mature F protein, which consists of two subunits (F1 and F2) required for the initial viral attachment and subsequent fusion peptide-mediated entry process [37,38]. Glycoproteins H and F interact with protein receptors in the host cell membrane determining host susceptibility, tissue tropism, and viral pathogenesis [39,40]. The H glycoprotein is a major determinant for virus–host interactions, being responsible for the virus attachment to the host cellular receptors, triggering the activation of glycoprotein F and fusion peptide exposure.

## 3. Genetic Heterogeneity of FeMVs

**Figure 1:**
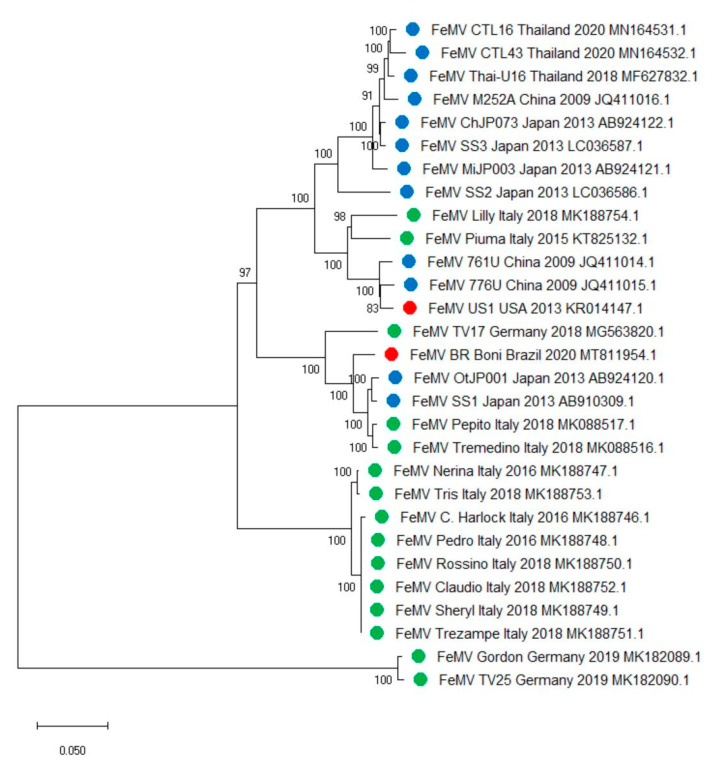
Phylogenetic analysis based on publicly available feline morbillivirus (FeMV) full-genome sequences. The tree with the highest log likelihood (−73307.55) is shown. The percentage of trees in which the associated taxa clustered together is shown next to the branches. The evolutionary history was inferred by using the Maximum Likelihood method and General Time Reversible model [43]. Initial tree(s) for the heuristic search were obtained automatically by applying Neighbor-Join and BioNJ algorithms to a matrix of pairwise distances estimated using the Maximum Composite Likelihood (MCL) approach and then selecting the topology with superior log likelihood value. A discrete Gamma distribution was used to model evolutionary rate differences among sites (five categories (+G, parameter = 0.5429)). The rate variation model allowed for some sites to be evolutionarily invariable ([+I], 30.85% sites). The tree is drawn to scale, with branch lengths measured in the number of substitutions per site. This analysis involved 29 nucleotide sequences. There was a total of 16,001 positions in the final dataset. Evolutionary analyses were conducted using MEGA X [44]. Legend: blue, FeMVs identified in Asia; green, FeMVs identified in Europe; red, FeMVs identified in America.

The FeMV strains are classified into two genotypes, with genotype 1 being the most prevalent worldwide (Figure 1). Genotype 1 was identified for the first time in domestic cats in Hong Kong; mostly urine samples, along with one rectal swab and one blood sample tested positive by RT-PCR amplifying a partial fragment of the L gene of morbilliviruses. The complete genome sequences of three FeMV strains showed nucleotide identities below 80% with other paramyxoviruses and were phylogenetically clustered with other morbilliviruses [6]. Additional evidence of the circulation of FeMV genotype 1 was found among domestic cats in Japan in 2014 [10,11,41]. The Japanese strains were genetically divergent, as they shared a nucleotide identity between 90.6% and 96.8% [11]. FeMVs genotype 1 were reported in cats from Malaysia [27] and Thailand [17], with strains from Malaysia showing a high nucleotide sequence identity (99%) with other Asian FeMVs (Thailand, Japan, and Hong Kong) [27]. Likewise, phylogenetic analysis of FeMV strains from Thailand revealed that they clustered with those reported in Hong Kong and Japan, sharing the highest nucleotide identity with FeMV strains SS3 and M252A (97.98–98.5% and 97.5–98.3%, respectively) [17]. More recently, additional FeMVs were identified and characterized from Mainland China [28]. Genetic analysis showed that the circulating FeMVs were most closely related to the Asian isolates Japan-N153U (99.4–99.6% nucleotide identity), Thailand-U16 (99.4–99.6% nucleotide identity), and Malaysia-PCS139 (99.6–99.8% nucleotide identity) and genetically diverse from isolates from other countries (91.8–96.1% nucleotide identity) [28]. Since 2015, FeMV was repeatedly detected in Europe, specifically in Germany, Italy, and Turkey [15,18,26]. In Italy, FeMV was described for the first time in 2015, and the whole genome sequence of the involved strain (Piuma/2015) was characterized [19]. Piuma/2015 showed the highest sequence nucleotide identity (94.5%) with early FeMV strains 761U and 776U from Hong Kong [6] and the lowest (88%) with strains OtJP001 and SS1 from Japan [11,41]. Upon sequence analysis based on the partial L gene sequences available for FeMVs from Europe, Piuma/2015 showed a nucleotide identity of 84.1–96.1% and 81.2% with German and Turkish FeMV sequences, respectively [19], highlighting the existence of genetic heterogeneity in Europe as already described for Japanese FeMVs [11]. Additionally, the complete genome of nine FeMVs and additional 27 partial L gene sequences detected in Italy were characterized as FeMVs genotype 1 clustering in two different clades. Sequences of the first clade bore the highest nucleotide sequence identity with Hong Kong FeMV strains 761U and 776U (95.4–97.3% and 94.3–96.2%, respectively) and with the first discovered Italian FeMV Piuma/2015 strain (95.4–100%), which shared only a 88.4–89.5% nucleotide identity with sequences belonging to the second clade [22]. The whole genome sequence of one FeMV strain identified in USA, referred to as FeMV^US1^, was shown to be 98% identical to the Hong Kong isolates 776U and 761U [14]. On the other hand, the one from Brazil (FeMV BR Boni), bore the highest nucleotide identity (97%) with the isolate Pepito/2018 from Italy and the Japanese strain SS1, while the nucleotide identity with FeMV^US1^ was 87.8% [16]. The phylogenetic analysis based on the 29 publicly available whole genome sequences suggests the existence of two different clades within FeMVs belonging to genotype 1, the first including FeMVs from China, Japan, Thailand, Germany, Italy, Brazil, and USA, and the second including only FeMVs from Italy. Additionally, within the first more numerically representative clade, three clusters may be distinguished, further evidencing FeMVs heterogeneity (Figure 1). A new genotype, thereby designated FeMV genotype 2 (FeMV-GT2), was discovered in Germany [42]. FeMVs belonging to genotype 1 and FeMV-GT2 share a genome nucleotide sequence identity of approximately 78.2% [42]. Little is known about the clinical significance of the genetic heterogeneity of FeMVs. It is thus important to investigate if any diversity in clinical outcome occurs within genetically divergent strains of the same genotype as well as between FeMV genotypes. 

## 4. Epidemiology: Known and Unknown

### 4.1. Prevalence of FeMV

The current literature on FeMV suggests that genotype 1 has a global distribution. Data on the molecular prevalence of FeMV are summarized in Table 1. Although studies vary considerably in size and origin of the enrolled cats, they document that FeMV RNA can be found mostly in urine and kidney tissues with a prevalence ranging from 3% [14] to 39.4% [27]. Interestingly, surveillance studies focusing on cats living in multi-cat environments showed higher infection rates than those from household cats [10,15,16,22]. This finding is likely due to the higher probability of successful transmission occurring in cats having multiple contacts with other cats. Intriguingly, a higher risk of FeMV infection was found in unneutered male cats compared to female cats [12,27]. Surveillance studies evaluating FeMV seroprevalence are summarized in Table 2. As observed in molecular studies, seroprevalence values vary widely, ranging from 17.32% [22] to 63% [45]. In a recent study conducted in Italy, molecular and serological prevalence were evaluated based on age. Prevalence of FeMV RNA was found higher in urine samples collected from young and middle-aged cats, while prevalence of FeMV antibodies was higher in old cats [22]. The molecular prevalence of FeMV-GT2 was as low as 0.83% in urine samples, which is lower than that obtained in reports on genotype 1 [25]. In the most recent study from Chile [45], 112 serum samples from domestic cats were serologically tested against FeMV genotype 1 and FeMV-2 using an indirect immunofluorescence (IIF) assay. Here, 63% of the samples showed antibodies against one of the 2 FeMV genotypes, while 30% of samples were seropositive for both genotypes. It is noteworthy that further serological studies can be informative in assessing the real global prevalence of FeMV. However, these studies might not be as informative on the FeMV genotype-specific prevalence, since cross-reactive antibodies may be present in FeMV-GT1- or -GT2-positive cats. Additionally, anti-CDV neutralizing antibodies have been reported in cats [46,47]. Interestingly, sera from dogs infected with CDV were demonstrated to cross-react with FeMV N protein and, in a similar way, sera from FeMV-infected cats cross-reacted with the N protein of CDV [11]. It is thus important to confirm these findings at a larger scale and whether genotype-specific antibodies are elicited following a primary FeMV infection.

### 4.2. FeMV Persistent Infection 

Numerous studies have reported evidence of chronic infection by FeMV. In one study, FeMV strain US1, identified from a domestic male cat in 2013, was detected in the same cat more than a year after the first RNA detection [14]. Viral RNA was constantly found up to day 110 in the first FeMV-positive cat from Italy [18,20] and up to 10 months after the initial molecular detection from five FeMV-positive carcasses [22]. Furthermore, virus shedding was observed for several months in urine samples from two cats infected with FeMV-GT2 [25]. These findings may suggest the capability of FeMV to establish a persistent infection, although this remains to be ascertained in an experimental setting. Although the mode of transmission of FeMV is currently unknown, the above observations suggest that FeMV might be present in infected cats for a long time, which is consistent with the high positive rates in cats living in colonies. Few investigators have successfully detected FeMV RNA from blood samples [6,10]. This may implicate that viremia occurs for a short period of time, or alternatively this may be related to the retrospective nature of the studies and to sample collection time, which likely occurred after the viremic phase.

## 5. FeMV as the Causative Agent of Renal Disease?

The role of FeMV in the pathogenesis of feline CKD is still debated. A case–control study proposed a link between FeMV infection and TIN [6], and several studies have been performed to ascertain the association between FeMV infection and feline CKD, leading to controversial conclusions [15,26,29]. An association between feline paramyxoviruses and CKD has been suggested in a systematic investigation involving domestic cats with and without CKD [26]. Paramyxoviral RNA was detected from samples of eight cats belonging to the CKD-affected group, while none of the urine samples of the control group was positive for paramyxoviral RNA. All cats were affected by lower urinary tract disease or renal disease at the time of sample collection [26]. CKD is a frequent clinical scenario with a described prevalence of 1–3% and a peak of 30% in elderly cats [48]. Consequently, a prompt CKD diagnosis is necessary to avoid complications and to decrease the degeneration of renal functionality in domestic cats [29]. The diagnosis of CKD and staging are mainly based on the concentration of serum creatinine, urine protein/creatinine ratio, urine concentrating ability, and diagnostic imaging [49]. These parameters, however, are late markers that are more indicative of a CKD diagnosis in advanced stages. Alternatively, and as indicated by the International Renal Interest Society guidelines (IRIS), (http://www.iris-kidney.com/guidelines/, accessed on 14 April 2021) serum symmetric dimethylarginine has been suggested as an early indicator of CKD. In addition, urinary qualitative proteinuria and electrolytes urinary fractional excretion have been considered in the assessment of renal damage [7,50]. Specifically, qualitative proteinuria and proteomics allow the analysis of biomarkers indicative of kidney damage such as cauxin, uromodulin, and retinol binding protein [50,51,52].

In this regard, clinical data from a cohort of 14 FeMV-infected cats were compared with data obtained from 22 healthy and 21 CKD cats [29]. CKD was diagnosed in three out of 14 FeMV-infected animals. Interestingly, even though only one cat was classified as proteinuric and five cats as borderline-proteinuric according to IRIS staging of CKD Guidelines (http://www.iris-kidney.com/guidelines/ accessed on 14 April 2021), sodium-dodecyl-sulphate-polyacrylamide gel electrophoresis (SDS-PAGE) analysis of urine samples revealed qualitative proteinuria in 77% of FeMV-infected cats. In these subjects, a tubular pattern characterized by a reduction in uromodulin and augmented low-molecular-weight proteins was observed. Infection with FeMV was therefore related to diverse levels of kidney impairment, varying from minor tubular proteinuria with less concentrated urine to azotemia. The data from this report highlight that this infection may be associated with the occurrence of a sub-clinical renal disfunction in younger cats as compared to those commonly presenting CKD [29]. 

Several studies have been conducted in cats to investigate the link between FeMV infection and TIN, which involves primary injury to renal tubules and interstitium and represents the most frequent histopathological finding in CKD. A case–control report described the occurrence of TIN in 7 out of 12 FeMV-positive cats and in only 2 out of 15 FeMV-negative cats [6]. Histological examination revealed interstitial inflammatory infiltrate and renal tubular degeneration or necrosis in kidney sections from positive cats. In addition, a marked decrease in cauxin expression in the degenerated tubular epithelial cells was observed. FeMV immunoreactivity was revealed within kidney tubular cells and lymph node-resident macrophages [6]. In a subsequent study, although no statistically significant relationship was confirmed between TIN and FeMV infection, a significant association was found between FeMV and the presence of renal inflammatory lesions [12]. Moderate to severe chronic interstitial nephritis, mild infiltration of inflammatory cells and other lesions, including interstitial fibrosis, glomerulosclerosis, tubular microcystic change, proteinaceous casts, and calcification were described in FeMV-positive kidney tissues. Here, FeMV immunoreactivity was limited to tubular epithelial cells of the renal cortex, medulla, and pelvis, and not detected in inflammatory cells [12]. To study the association between FeMV infection and pathological changes in kidney tissues of infected cats, 38 kidney tissue samples were evaluated using immunohistochemistry and immunofluorescent assays [13]. Certain tissue damage scores were statistically higher where FeMV antigen was detected, particularly those associated with renal tubular tissues. The histopathological findings correlated with the presence of FeMV antigens were tubular atrophy, fibrosis, interstitial cell infiltration, and glomerulosclerosis. Feline IgG were also found in glomerular tissues. However, a different localization as well as the absence of a significant association with the FeMV antigens was reported [13]. On the other hand, different studies failed to report a statistically significant association with kidney disease [10,15,16,17,21,22,23,24,28]. Several reasons can explain these controversial findings. First, the retrospective nature of the studies does not allow to establish a definite association between FeMV infection and kidney pathological lesions or CKD. In addition, given the general complexity of CKD pathogenesis, it is difficult to associate CKD to a single etiological trigger in cats. In fact, in most cats, the underlying etiology of CKD is not completely understood. Various factors potentially contributing to renal damage have been proposed, including toxic insults, hypoxia, chronic glomerulonephritis, chronic pyelonephritis, upper urinary tract obstructions, and viral infections such as feline immunodeficiency virus, feline infectious peritonitis virus, and feline leukemia virus [53,54,55,56,57]. Therefore, further efforts are warranted to disentangle the role of FeMV in comorbidities and clarify whether FeMV infections are causatively involved in feline CKD or just benefit from inflamed tissues of the upper urinary tract. Also, certain feline chronic diseases such as TIN, may still progress when FeMV is not molecularly detected in urine or FeMV antigens in the lesions might have been already eliminated by the host immune response in the case of severe chronic TIN, a phenomenon already described for other viral infectious diseases [58,59]. Since a definite role of FeMV in the pathogenesis of renal disease is difficult to ascertain, given the retrospective nature of these studies, it is clear that in vivo experimental infection is needed to examine the pathogenesis of FeMV and the elicited immune response during the acute and chronic stages of infection.

## 6. FeMV: Not Just a Renal Pathogen?

Morbilliviruses related to FeMV, such as CDV and MeV, invade the host following aerosol infection [60]. After a primary replication in dendritic cells and tissue macrophages located in the draining lymph nodes, they can infect lymphocytes, resulting in a short-term viremia [61], which leads to viral spread to the other organs. Infection by morbilliviruses has been described in various tissues such as the lung, kidney, gastrointestinal tract, vascular endothelium, and brain [33]; however, the detailed cell entry mechanisms of FeMV are unknown. The signaling lymphocyte activation molecule (SLAM or CD150) and Nectin-4 are potential candidates since they represent the major receptors for other morbilliviruses in immune and polarized epithelial cells, respectively [40,62,63,64]. In this regard, FeMV has been observed to infect in vitro diverse feline cell lines such as epithelial, fibroblastic, lymphoid, and glial cells [65]. These findings suggest that the receptor(s) of FeMV may be expressed in multiple tissues. FeMV F protein has only one basic proteolytic cleavage site, while the cleavage sites in other morbilliviruses are multibasic [6]. Based on the amino acid differences of other morbilliviruses, this observation suggests that the F protein of FeMV may be cleaved by different proteases, thus affecting viral entry and host cell tropism. Indeed, contrarily to F proteins containing multibasic cleavage sites which are targeted by ubiquitous endopeptidases (e.g., furin), F proteins with a single basic residue at the cleavage site may be cleaved by substrate-specific extracellular proteases (such as trypsin-like enzymes), as reported for other paramyxoviruses [66]. 

The in vitro tropism of FeMV-GT2 was investigated on primary cells from cat kidney, urinary bladder, lung, peripheral blood mononuclear cells, and brain [25]. The main target of FeMV-GT2 infection was renal epithelial cells, whereas epithelial cells from the urinary bladder were less susceptible to infection. Feline lung epithelial cells and cerebrum- and cerebellum-derived cells were described to be susceptible to FeMV-GT2 infection. In addition, FeMV-GT2 was demonstrated to be able to infect numerous immune cells such as monocytes, B lymphocytes, CD4^+^ T lymphocytes and to a lesser extent, CD8^+^ T lymphocytes. However, no cytopathic effect (CPE) was detected in any of the tested cell lines, except for cerebellum cultures from which small syncytia were observed [25]. This is in contrast with previous reports on FeMV genotype 1 which induced syncytia formation in Crandell–Rees feline kidney (CrFK) [11] and feline embryonic fibroblast (FEA) cells [22,67]. 

In a subsequent study, the in vitro tropism was investigated by virus histochemistry (VHC) using FeMV genotype 1. VHC revealed specific immunoreactivity in lungs, kidneys, and brain sections, with FeMV particles being able to bind to epithelial cells of bronchioles and alveolar macrophages in the lungs. In kidneys, FeMV antigens were found in inflammatory cells located in the lumen of tubuli and in glomeruli. In brain tissues, immunoreactivity was weak and occasionally seen in cerebellar granule cells and in inflammatory cells within blood vessels [22]. Detection of FeMV RNA in diverse extra-renal tissues such as lung, spleen, liver, lymph node, and brain has also been described in naturally infected cats. Severe cholangiohepatitis and splenic hyalinosis, megakariocytosis with lymphocytic depletion were described in RNA-positive tissues, and FeMV immunoreactivity was observed within the cytoplasm of hepatocytes and mononuclear cells, respectively [15,20,22]. The in vitro tropism and histopathological findings described above may suggest a role of FeMV in pathological processes, not only at the urinary tract level, but also in other organs.

## 7. FeMV Detection Methods

Even though virus isolation remains the reference standard for the diagnosis of morbilliviruses, it has been generally confirmed to be difficult and time-consuming for FeMV [6,11,22,25]. Molecular detection approaches followed by sequencing have been proven essential for a rapid confirmation of FeMV infection and to characterize new strains [11,22]. Serological studies have also been useful for the evaluation of FeMV epidemiology and to assess the immune status of feline populations in Italy, United Kingdom, Japan, China, and Chile [6,11,12,22,24,45,68]. However, it is important to mention that a commercial detection assay for FeMV is not currently available and it is not recommended, given the yet not completely clear pathogenetic role of FeMV infection in cats.

### 7.1. Virus Isolation

FeMV isolation has been achieved using urine samples of domestic cats inoculated into monolayers of CRFK cells [6,11]. African green monkey cells and diverse feline cell lines (epithelial, fibroblastic, lymphoid, and glial cells) have also been proven to be useful for the isolation of FeMV [69]. Typically, several weeks are needed before virus growth in the form of cell rounding, cell lysis, and syncytia formation [6]. Recently, FEA cells were shown to reduce from weeks to days the time necessary for FeMV isolation using urine diluted with Minimum Essential Medium Eagle immediately after collection [67]. Although an identical procedure was used in a study conducted by the same group [22], these results were not confirmed, and CPE was observed only after two 10-day periods of blind passage on FEA cells. This apparent inefficiency for rapid virus isolation might depend on either the biological characteristics of the virus, possibly combined with the absence of a proper viable cell line, or the timing of sample collection as well as inappropriate sample storage. A Vero cell line stably expressing the canine SLAM was shown to greatly improve the efficiency of isolation and propagation of CDV strains [70]. Therefore, it would be beneficial to establish a homologous cell system stably expressing the feline SLAM to attempt the rapid isolation and propagation of FeMV RNA-positive samples.

### 7.2. Reverse-Transcription Polymerase Chain Reaction

A primer set based on a 155-nt highly conserved region of morbillivirus L gene has been successfully used to detect FeMV by RT-PCR (reverse-transcription polymerase chain reaction) [6]. Using a similar approach, Furuya et al. designed a protocol that allows the detection of degraded RNA in formalin-fixed paraffin-embedded samples by amplifying a 115-nt region of the FeMV L gene [10]. In addition, a loop-mediated isothermal amplification technique targeting a 215-nt region of the FeMV L gene was developed for the detection of FeMV RNA [71]. Since then, other RT-PCR assays were also successfully used for FeMV diagnosis, including a pan-paramyxovirus primer set amplifying a 398-nt region of the L gene [14], a primer set for a 398-nt region of the N gene [27], and primers amplifying a 401-nt region of the L gene [22]. A quantitative RT-PCR, named qPCR_FeMV_, targeting a 76-nt region of the P/V/C gene, was developed for rapid detection and quantitation of FeMV RNA [20]. qPCR_FeMV_ was shown to have a higher sensitivity with respect to RT-PCR, enabling the detection of FeMV RNA even in samples with a low viral concentration. FeMV-GT2 RNA was first detected using a set of degenerated consensus primers able to detect known and novel paramyxoviruses [72]. The development of a quantitative RT-PCR assay specific for FeMV-GT2 may be useful to provide a more rapid differentiation between the two FeMV genotypes.

### 7.3. Serology

Various serological methods have been used to detect FeMV infection. However, a virus neutralization assay, the reference standard for diagnostic serology, has not been developed yet. Western blot, IIF, and an indirect enzyme-linked immunosorbent assay (iELISA) have been the main published platforms used to detect FeMV-specific antibodies. Western blot and IIF assays allowed the detection of antibodies directed against the N protein [6], whereas iELISA was developed to detect those against the P protein [68]. The N and P proteins are highly expressed in morbillivirus-infected cells, representing a good target for detecting the antibody response to FeMV [73]. Western blot analyses used FeMV isolate [11] or the recombinant N protein [6,24] as antigens. Feline antibodies were detected using a horseradish peroxidase-conjugated goat anti-cat antibody which reacts specifically with cat IgG and with light chains common to other cat immunoglobulins. For antibody detection by IIF, the N protein was expressed in HeLa cells, and antibody binding was detected using a goat anti-cat-IgG conjugated with fluorescein (FITC) specific for the IgG Fc region [12,22]. The iELISA used the recombinant P protein as antigen, and FeMV antibodies were detected using a horseradish peroxidase-conjugated goat anti-cat antibody [68]. An iELISA for the P protein has been described for other paramyxoviruses and showed an increased specificity and accuracy in comparison to other common assays [74]. It is noteworthy that iELISA tools may be useful in the diagnosis of FeMV-infected cats or may contribute as serological screening tools in epidemiological studies.

## 8. Conclusions

Cats are among the most common pets, and kidney failure represents one of the most important and frequent clinical scenarios, with CKD prevalence increasing with age, affecting up to 30% of cats older than 15 years [48]. Therefore, it is important to clarify the characteristics and the pathogenicity of FeMV in domestic cats as a natural host. Even if the association between FeMV and renal disorders has not been well defined, FeMV infection could lead to a potential renal insult causing an initial kidney disorder. FeMV has some distinctive characteristics, and the biology of FeMV, including its pathogenicity, is still not well understood partly due to its uniqueness. The described in vitro studies on FeMV tropism and extra-renal histopathological findings resemble the pathology of other morbilliviruses such as CDV or MeV and may suggest a role of FeMV in pathological processes in other organs besides the urinary tract. Since a well-defined etiopathogenetic role of FeMV cannot be ascertained in the case of a spontaneous disease due to the presence of other possible confounding factors, an experimental study in the natural host or in a susceptible animal model is highly needed.

## Figures and Tables

**Table 1:** Reported FeMV molecular prevalence of cats in relation to population sampled, type of sample, and geography.

Reference	Population	Total	Sample	FeMV RNA-Positive Tissues	Prevalence per Type of Sample	Prevalence ^c^	Country
**Woo et al., 2012**	Stray cats	457	Urine		11.6%	12.3%	Hong Kong
			Blood		0.2%		
			Feces		0.8%		
		16	Oral swab		6.2%	6.2%	Mainland China
			Rectal swab		6.2%		
**Furuya et al., 2014**	Household cats	82	Urine		6.1%	9.8%	Japan
		10	Blood		10%		
		10	Tissues	Kidney	40%		
**Sakaguchi et al., 2014**	Household cats	13	Urine		23%	23%	Japan
**Furuya et al., 2016**	Household cats	166	Urine		15.1%	15.1%	Japan
**Sharp et al., 2016**	Household cats	327	Urine		3%	3%	USA
**Park et al., 2016**	Stray/household cats	100	Urine		17%	22%	Japan
			Tissues	Kidney	18%		
**Darold et al., 2017**	Colony cats ^a^	17	Urine		52.9%	23%	Brazil
	Household cats	35			8.6%		
**Yilmaz et al., 2017**	Household cats	96	Urine		3.1%	5.4%	Turkey
		15	Tissues	Kidney	26%		
				Lymph nodes	13%		
				Lung	6%		
				Spleen	6%		
				Intestine	6%		
				Liver	6%		
**McCallum et al., 2017**	Household cats	40	Urine		12.5%	12.5%	United Kingdom
**Stranieri et al., 2019**	Stray cats	6	Urine		16.6%	3.2%	Italy
	Household cats	59			0%		
	Stray/household cats	27	Tissues	Kidney	7.4%		
**Mohd et al., 2019**	Stray/household cats	124	Urine		50.8%	39.4%	Malaysia
		25	Tissues	Kidney	80%		
**Sieg et al., 2019**	na	723	Urine		0.8%	0.83%	Germany
**De Luca et al., 2020**	Colony cats ^b^	69	Urine		31.8%	16.8%	Italy
	Household cats	127			8.6%		
	Colony cats ^b^	7	Tissues	Kidney	57.1%	22.8%	
				Bladder	14.2%		
				Spleen	28.5%		
				Lymph nodes	14.2%		
	Household cats	28	Tissues	Kidney	10.7%		
				Bladder	10.7%		
				Spleen	3.5%		
				Brain	3.5%		
**Muratore et al., 2020**	Household cats	127	Urine		3.9%	7.3%	
	Colony cats ^b^	40	Tissues	Kidney	7.5%		
	Household cats	23	Urine		26%	8%	
	Colony cats ^b^	10	Tissues	Kidney	10%		
**Ou et al., 2020**	n.a.	64	Urine		9.3%	9.37%	Mainland China
**Chaiyasak et al., 2020**	Colony cats ^b^	31	Urine		19.3%	11.9%	Thailand
	Household cats	100	Urine		13%		
	Colony cats ^b^	61	Blood		19.6%		
	Household cats	200	Blood		0%		

n.a., information about the origin of the cats was not available; ^a^ cats living in a multi-cat house; ^b^ stray cats; ^c^ cats with positive results in multiple tissues and/or samples were counted once

**Table 2:** Reported FeMV seroprevalence of cats in relation to population sampled and geography.

Reference	Population	Total	Seroprevalence	Country
**Woo et al., 2012**	Stray cats	457	27.8%	China
**Sakaguchi et al., 2014**	Household cats	13	23%	Japan
**Park et al., 2016**	Stray and household cats	100	21%	Japan
**Arikawa et al., 2017**	n.a.	100	22%	Japan
**McCallum et al., 2017**	Household cats	72	31%	United Kingdom
**De Luca et al., 2020**	Colony cats ^a^	69	21.73%	Italy
	Household cats	127	17.32%	
**Busch et al., 2020**	Colony cats ^a^	112	63%	Chile

n.a., information about the origin of the cats was not available; ^a^ stray cats.

## Data Availability

Not applicable.
